# Perceived Causes of Sleep Problems in Higher Education Students: An Exploratory Study

**DOI:** 10.1177/00332941241299730

**Published:** 2024-11-09

**Authors:** Daniel Ruivo Marques, Ana Allen Gomes, Maria Helena Pinto de Azevedo

**Affiliations:** Department of Education and Psychology, 451616 University of Aveiro, Aveiro, Portugal; CINEICC - Center for Research in Neuropsychology and Cognitive Behavioral Intervention, Faculty of Psychology and Educational Sciences, 37829 University of Coimbra, Coimbra, Portugal; Faculty of Psychology and Educational Sciences, 37829 University of Coimbra, Coimbra, Portugal; CINEICC - Center for Research in Neuropsychology and Cognitive Behavioral Intervention, Faculty of Psychology and Educational Sciences, 37829 University of Coimbra, Coimbra, Portugal; Faculty of Medicine, 37829 University of Coimbra, Coimbra, Portugal

**Keywords:** Sleep, insomnia, perception, causes, students, higher education

## Abstract

The major purpose of the present study was to explore higher education students’ perceptions of the causes of their sleep problems. The data were drawn from a previous cross-sectional online survey, from which only the questions relevant to this study were selected, including items on sleep disturbances and their causes. The participants included 1519 full-time students (76% women), aged 18–30 years (*M* = 20.9, *SD* = 2.3); 95.3% were single and studying for a Bachelor’s degree (75.2%) across diverse fields of study. Overall, 30% of the students reported suffering from insomnia, 17.8% met the criteria for clinical insomnia (according to the Insomnia Severity Index), and 12.5% reported being dissatisfied with their sleep. The most significant perceived causes of sleep difficulties were stress (85%), academic problems (67%), and general worries (56.5%). Other causes included affective/love problems (28.4%), family issues (27.5%), changes in sleeping habits (25.9%), financial problems (13.7%), transition to higher education (10.4%), pain (7.1%), and general illness (5.5%). These findings may have important implications for interventions aimed at improving sleep health among higher education students.

## Introduction

Higher education students are at high risk for disturbed sleep and their sleep health has become an issue of growing concern. Sleeping problems, including insomnia, are highly prevalent in this population at a global scale and are considered an important public health issue. In the evidence map by Bjørnnes et al. (2021), which includes studies on higher education students across six continents, the reported global proportion of sleep problems was 48%, ranging from 40% in Europe to 55% in Africa and South America. In a systematic review that included seven studies, the prevalence of insomnia in university students ranged from 9.4% to 38.2%, with a weighted mean prevalence of 18.5%, a much higher figure than the rates (7.4%) reported in the general population (Jiang et al., 2015).

According to a Norwegian national study focused on higher education students, the overall prevalence of insomnia (DSM-5 criteria) was 30.5% (Sivertsen et al., 2019). The authors also found that there was a substantial increase in sleep problems from 2010 (22.6%) to 2018 (30.5%), which was especially pronounced in women. It is also of note that, for many students, sleep complaints are persistent over time (Azevedo et al., 2010), and a recent study showed that childhood-onset insomnia symptoms (40%) persist through young adulthood (Fernandez-Mendoza et al., 2022). In a previous study using the complete sample from which the current sample was selected for analysis in this study (cf. Material and Methods), about 26% participants reported having sleep difficulties “since they can remember”, and 26% indicated that their sleeping difficulties had lasted “for more than one year” (Silva et al., 2021).

The transition from high school into and through higher education represents a life stage in which many students undergo various changes as well as potential challenges (Arnett et al., 2014).

There is strong evidence that stress plays a key role in the development of sleep problems in higher education students (Gardani & Bradford, 2022). In a large sample of undergraduate students, the tension-anxiety dimension of the Profile of Mood States (POMS) and perceived stress were the most important factors in predicting poor sleep quality (Lund et al., 2010).

A study conducted by Cabrita et al. (2004), based in a large sample of Lisbon University (Portugal) students, found that complaints of anxiety, depression and insomnia were the most frequently stated health problems to justify the use of psychoactive medicines. The prevalence of use was significantly higher in those who perceived their health status as weak due to intense stress.

Regarding the frequency of using sleep aids in the already mentioned study by Silva et al. (2021), students reported using them “only in stressful situations/when stressed” with about 23% using prescribed sleep medications and about 33% using over-the-counter/supplements.

Students in higher education institutions face a broad range of ongoing stressors related to academic issues (e.g., classroom presentations, lack of time to complete assignments, academic overload, examinations) that impact students’ learning capacity, academic performance, and mental health problems, such as depression, anxiety, and sleep disturbances (cf. García-Ros et al., 2012; Pascoe et al., 2020). In the study by Lund et al. (2010), students overwhelmingly stated that emotional and academic stress negatively affected sleep.

In this context, it may be worth noting that the most frequent kind of worry reported by Portuguese university students (42%) was related to university issues (e.g., academic performance, future expectations, and personal and professional success), followed by worries about time management/organization of daily activities (38%) including academic and leisure activities, and having no free time (Reis et al., 2019).

Academic stress has become a widespread problem worldwide, and may be the single most dominant stress factor that affects the well-being and academic success of students in a serious way: “*Last year… I almost always felt stressed out… I had many subjects. All of them were very demanding. Intense anxiety, worrying and insomnia seriously damaged my academic functioning*” (undergraduate patient repeating their second school year).

In the study by Reis et al. (2019), issues related to economic/financial matters were the third most frequent kind of worry (22%). Another study found that stress related to financial situation was reported by 68.6% of students (Karyotaki et al., 2020). Sleep disturbances are also found in students experiencing stress related to financial stress/strain (Galambos et al., 2009; Peltz et al., 2021).

Research on the links between family and romantic relationships and sleep quality among higher education students is very limited (Adams et al., 2014; Bajoghli et al., 2014). This is significant given that a large number of undergraduates experience stress related to their own romantic lives (67%) and report feeling stressed due to problems faced by loved ones (75%) (Karyotaki et al., 2020). Furthermore, among Portuguese university students, the fourth most frequent worry reported (16%) was related to family matters such as general family life and issues like illnesses and deaths (Reis et al., 2019).

According to Adams et al. (2014), insecure and especially anxious attachment relationships are associated with sleep problems in young children and adults (Adams et al., 2014; Bajoghli et al., 2014). Among university students in romantic relationships, poor sleep quality was found among those subjects with higher scores in obsessive traits, and with a preoccupied, fearful, or dismissive attachment style (Talero-Gutierrez et al., 2017).

Love and family life are complex issues that can affect individuals sleep either positive or negatively (Adams et al., 2014; Bajoghli et al., 2014).

Health can also be a source of stress/worries. About 64% of undergraduates experienced at least mild stress about their health (Karyotaki et al., 2020) and 12% of university Portuguese students reported worrying about personal issues including wellness and health, unwellness and potential health compromising behaviours (Reis et al., 2019). In a large sample of higher education students, insomnia was found to be strongly associated with many physical problems, but especially with mental conditions (Sivertsen et al., 2021).

Transition to higher education represents a big life change, experienced by many students as highly stressful, a loss. In fact, for some, it is a dramatic shift in their lives: “*… going to university and being far from home and my family is still one of the worst things that has ever happened to me. When I finished school and moved far away from home to study, I cried every day …slept poorly… felt sad, lonely…* (undergraduate patient).

The phenomenon of irregular sleep patterns is well known among university students. Sleep-wake behaviour may be one of the first daily habits that change in the transition from high school to university (Gomes, 2005; Lund et al., 2010; Tsai & Li, 2004). According to Gomes et al. (2009), compared to high school, 35% of first-year students reported that since entering university their sleeping habits had changed a lot; this figure increased to 52% in third year students. In a study on college students, improper sleep scheduling was found to increase the risk of insomnia symptoms (Gellis et al., 2014). In the already mentioned study by Silva et al. (2021), 20.4% of students reported having sleep difficulties upon entering higher education. Irregularities/daily intraindividual variability of sleep/wake patterns in the general population were associated with adverse health outcomes, including depression symptoms, stress, symptoms of insomnia and poor sleep (cf. Bei et al., 2016).

Despite the increasing interest in students᾽ sleep health in recent years, there is a lack of research on their perspectives about the factors that might cause their sleep problems (Bjørnnes et al., 2021; Robotham, 2008; Zhou et al., 2022).

Indeed, there is evidence that better health outcomes are obtained when the communication between doctor and patient leads to agreement on problem and problem solving (Stewart, 1995). As highlighted in the systematic review by Araújo et al. (2017), there is a mismatch between patients’ and health care professionals’ points of view on insomnia, and that mismatch contributes to patient sense of frustration, conflict and misunderstanding. Health care professionals tend to give little attention on the patient’s subjective experience of insomnia. Additionally, the results may be used to assist higher education institutions in developing prevention and intervention programs to improve students’ sleep health, and their wellbeing and academic success.

To the best of our knowledge, only two studies have examined this topic in the general population (Armstrong & Dregan, 2014; Petersen et al., 2022). Notwithstanding their obvious relevance, it is important to consider the target population of higher education students by including factors that are more applicable and meaningful to their lives. To date, no previous research has investigated this issue among Portuguese students. Based on the above, the primary aim of this study is to explore higher education students’ perceptions of the causes of their sleep problems. Additionally, we examine the distribution of perceived causes of sleep difficulties across different demographics, as well as various sleep and psychological factors.

## Materials and Methods

### Participants

A total of 2029 Portuguese students aged 18–78 years old completed the survey. To be included in the present study, participants had to be full-time students (the typical situation in Portugal) and aged between 18 and 30 years, to minimize the effects of extracurricular activities and comorbidities. From a total of 2029 of students who completed the online survey, 1519 (74.86%) met the inclusion criteria and were included in the present study. It is important to note that the current study is based upon an existing larger database (e.g., Silva et al., 2021).

### Measures

For the present study purpose, the following variables were selected:

*Socio-demographics*: age, sex, marital status; cycle of studies; and field of study.
*Sleep related variables*: Current insomnia was measured by a single yes/no question: “*Do you suffer from insomnia currently?*”. Sleep (dis)satisfaction was evaluated with item 2 from the Insomnia Severity Index (Morin et al., 2011): “*How satisfied/dissatisfied are you with your current sleep pattern?*” whose response options are: Very Satisfied = 0, Satisfied = 1, Moderately Satisfied = 2, Dissatisfied = 3, Very Dissatisfied = 4. In the DSM-5, a complaint of dissatisfaction with sleep is an essential feature of insomnia disorder (see introduction) and it seems to be a better indicator of sleep pathology than criteria used by classifications systems such as the DSM-IV and the ICSD (Ohayon & Reynolds, 2009). In addition, sleep satisfaction is an important indicator of sleep health (Buysse, 2014). Perceived cause(s) of sleeping difficulties was evaluated with the item “*In your opinion, what is(are) the cause(s) of your sleeping difficulty(ies)? From the list below, choose the answer(s) you consider most important”*. The list covered the following topics: stress, academic/study problems (e.g., work load, exams, presentations, evaluations), family problems, affective/love problems; financial problems; worries in general; illness in general; pain; change in sleeping habits; transition to higher education; other cause(s); I don’t know). The options provided were chosen based on literature and clinical experience with many young individuals in an outpatient clinic at a University Hospital.

#### Insomnia Severity Index (ISI)

The European Portuguese version of the ISI was used to assess the subjective severity of insomnia (Clemente et al., 2021; Morin et al., 2011). This instrument consists of seven items, four of which related to the night symptoms and three regarding the day. The items are summed to obtain a total score that may range between 0 and 28 points. Higher scores denote greater insomnia severity. A score ≤7 indicates no insomnia, 8–14 indicates subthreshold clinical insomnia, 15–21 indicates clinical insomnia of moderate severity, and a score of 22–28 indicates severe insomnia (Morin et al., 2011). In the present study, Cronbach᾽s *α* was .82.

#### Hospital Anxiety and Depression Scale (HADS)

The HADS is a self-report scale designed to assess anxiety (subscale HADS-A, 7 items) and depression (subscale HADS-D, 7 items) and for each subscale the score is the sum of the respective 7 items (ranging from 0 to 21). The total score is the sum of the 14 items, with a possible range from 0 to 42. A higher score represents higher levels of anxiety and depression. A score of 7 or lower indicates no symptoms of anxiety or depression, a score between 8 and 10 suggests mild anxiety or depression, between 11 and 14 moderate anxiety or depression and between 15 to 21 anxiety or depression. A score of ≥11 is considered clinically significant anxiety or depression (Zigmond & Snaith, 1983; Pais-Ribeiro et al. (2007). The HADS was found to perform well in assessing the severity of symptoms and caseness of anxiety and depression disorders in both clinical settings and the general population (Bjelland et al., 2002; Snaith, 2003). In the present study, we used the European Portuguese version of the HADS developed by Pais-Ribeiro et al. (2007), and the Cronbach᾽s *α* for HADS-A and HADS-D were .85 and .76, respectively.

### Procedure

Data for this cross-sectional survey study were collected through an online platform (Questionnaires UA) that was available for 32 days (from 28 February 2021 to 31 March 2021). Participants were required to declare their Portuguese nationality and voluntary participation, and to be enrolled in higher education institutions (Undergraduate, Master’s Degree, PhD) and aged 18 years or older (for details, see Silva et al., 2021). The Ethics and Deontology Committee of the university where the first author belongs approved the study. Higher Education institutions from all over the country (public and private) were invited to share the survey link to their students. In addition, we also recurred to dissemination through digital social networks such as Facebook, Linkedin, and Instagram.

### Statistical Analysis

Frequencies, contingency chi-square test, means, standard deviations and ranges were computed through the IBM SPSS Statistics v. 29 software.

To examine the most frequent combinations of sleeping difficulties causes, we used the iNZight 4.3.0 (https://inzight.nz/) software, specifically the module for analyzing multiple-response type data.

## Results

### Sample Characteristics

The sample comprised 1519 full-time students, including 1168 women (76%) and 351 men (23.1%), aged 18–30 years (*M* = 20.90, *SD* = 2.32). Of these, 48.7% (*n* = 739) were aged 18–20 years, 43.3% (*n* = 658) were aged 21–24 years, and 8% (*n* = 122) were aged 25–30 years. The great majority were single (*n* = 1447; 95.3%), 1.2% (*n* = 18) married or cohabiting and other 3.6% (*n* = 54) were divorced, separated, or widowed. Most of the students were studying for a Bachelor’s degree (75.2%, *n* = 1143), 22.5% (*n* = 342) for a Master’s degree, and 2.2% (*n* = 34) for a PhD. They were attending the following fields: 27.2% (*n* = 413) in Sciences and Engineering, 25.9% (*n* = 394) in Social Sciences and Humanities, 13.4% (*n* = 204) in Health and Biomedical Sciences, 13.0% (*n* = 197) in Arts, 10.1% (*n* = 153) in Behavioural Sciences, 8.4% (*n* = 128) in Languages and Literature, and 2.0% (*n* = 30) in “Other”.

### Descriptive Statistics

Means (*M*), Standard Deviations (*SD*) and range for all participants (*N* = 1519) were: Sleep satisfaction total score (*M* = 2.02; *SD* = 1.06; *Min* = 0; *Max* = 4); ISI total score: (*M* = 9.49; *SD* = 5.15; *Min* = 0; *Max* = 24); HADS total score (*M* = 13.8; *SD* = 7.16; *Min* = 0; *Max* = 35); HADS-A (*M* = 8.38; *SD* = 4.44; *Min* = 0; *Max* = 21); and HADS-D (*M* = 5.42; *SD* = 3.56; *Min* = 0; *Max* = 18). In total, 30% of students (*n* = 456) reported currently suffering from insomnia.

Clinically significant insomnia was present in 17.8% (*n* = 270) of which 16.5% (*n* = 250) had moderate insomnia and 1.3% (*n* = 20) had severe insomnia; 43.8% had subthreshold insomnia (*n* = 665) and 38.4% (*n* = 584) had no insomnia. A higher proportion of women reported more sleep disturbance compared to men (χ^2^_(1)_ = 15.745; *p* < .001; Crámer´s *V* = .10). The majority of the students were satisfied (Very Satisfied + Satisfied) with their sleep, with 87.6% (*n* = 1330) reporting satisfaction. Conversely, 12.5% of students reported sleep dissatisfaction (Moderately Satisfied + Dissatisfied + Very Dissatisfied), of which 9.9% (*n* = 150) were moderately satisfied and 2.6% (*n* = 39) were dissatisfied (Dissatisfied + Very Dissatisfied). According to the HADS, 38.9% of students experienced clinically significant anxiety (HADS-A ≥11, *n* = 484), of which 21.8% (*n* = 331) had moderate anxiety and 10.0% (*n* = 153) had severe anxiety. Additionally, 22.2% (*n* = 338) had mild anxiety, while 45.9% (*n* = 697) reported no symptoms of anxiety. With regard to depression, 9.3% (*n* = 142) had clinically significant depression (HADS-D ≥11), with 8.4% (*n* = 127) experiencing moderate depression, 0.9% (*n* = 15) severe depression, and 18.5% (*n* = 281) mild depression. Furthermore, 72.1% (*n* = 1096) reported no symptoms of depression.

### Perceived Cause of Sleeping Difficulties

Out of the ten options provided, three were considered the most important by participants: stress (85%, *n* = 399), academic problems (67%, *n* = 302), and worries in general (56.5%, *n* = 255) were by far the most frequent options. Affective/love problems, family problems and changes in sleeping habits were selected by similar percentages of respondents: 28.4% (*n* = 128), 27.5% (*n* = 124) and 25.9% (*n* = 117), respectively. Financial problems were selected by 13.7% (*n* = 62) and higher education transition by 10.4% (*n* = 47). Pain 7.1% (*n* = 32) and illness in general 5.5% (*n* = 25) were the least frequent options; 9.3% (*n* = 42) of individuals did not manage to identify any specific causes for their sleeping problems. None of the students provided a written answer to the open question: “other cause(s), please describe”.

Table 1 lists perceived causes of sleeping difficulties by sex. On average, both men and women selected three reasons for their sleeping problems. The most common combinations for men were “stress” (*n* = 10), “stress + family problems” (*n* = 9), and “stress + family problems + financial problems” (*n* = 8). Concerning women, the most common combinations were “stress + family problems” (*n* = 64), “stress + academic problems + family problems” (*n* = 56), and “stress” (*n* = 44). As for men and women together, the most common combinations were “stress + family problems” (*n* = 69), “stress + academic problems + family problems” (*n* = 61), and “stress” (*n* = 54).

**Table 1.** Perceived Causes for Sleep Difficulties by Sex (*n* = 456).

	Men *n* (%)	Women *n* (%)
Total	75 (16.4)	381 (83.6)
Stress	60 (82.2)	339 (89.7)
Academic problems	44 (60.3)	258 (68.3)
Family problems	14 (19.2)	110 (29.1)
Affective/love problems	24 (32.9)	104 (27.5)
Financial problems	13 (17.8)	49 (13.0)
Worries in general	33 (45.2)	222 (58.7)
Illness in general	6 (8.2)	19 (5.0)
Pain	3 (4.1)	29 (7.7)
Sleeping habits change	26 (35.6)	91 (24.1)
Higher education transition	8 (11.0)	39 (10.3)
Other causes	0 (0)	0 (0)
Do not know	11 (15.1)	31 (8.2)
Missing	2 (2.7)	3 (0.8)

*Note.* The participants could choose more than one option.

As observed in Table 1, a higher proportion of women identified stress (89.7%), academic problems (68.3%), worries in general (58.7%), family problems (29.1%), and pain (7.7%) as the main causes for their sleeping difficulties, whereas most men selected affective/love problems (32.9%), financial problems (17.8%), illness in general (8.2%), change in sleeping habits (35.6%), and “I don’t know” (15.1%). The figures for the transition to higher education were similar for both sexes: 11% for men and 10.3% for women.

### Perceived Causes for Sleep Difficulties by Age Groups

As can be observed in Table 2, there is a decrease in the perceived causes across age groups, particularly for “worries in general”, which decreased from 53.3% in the 18–20 age group to 9% in the 25–30 age group. Table 2 also shows that the proportion of all perceived causes is higher in the 18–20 and 21–24 age groups compared to the 25–30 age group. The highest overall percentage is 53.3% for “worries in general” in the 18–20 age group, significantly higher than the highest percentage across the various reasons for the 25–30 age group (10% for “illness + pain”).

**Table 2.** Perceived Causes for Sleep Difficulties by Age Groups (*N* = 456^a^).

	18–20 Years *N* (%)	21–24 Years *N* (%)	25–30 Years *N* (%)
Stress + academic problems	198 (47.7)	181 (43.6)	36 (8.7)
Family problems + affective/love problems + financial problems	100 (48.3)	89 (43.0)	18 (8.7)
Worries	136 (53.3)	96 (37.6)	23 (9.0)
Illness + pain	24 (48.0)	21 (42.0)	5 (10.0)
Sleeping habits change + higher education transition	69 (48.3)	68 (42.0)	6 (4.2)

*Note.* The participants could choose more than one option.

^a^It includes 5 (1.1%) missing values from participants who self-reported sleeping problems but did not choose any of the options.

### Perceived Causes for Sleep Difficulties by Cycle of Studies

As shown in Table 3, the percentages of perceived causes are significantly higher among Bachelor students compared to Master and PhD students. Regarding the highest percentages, 85.3% of Bachelor students selected “sleeping habits change + Higher Education transition”, while 23.7% of Master and PhD students selected “family + love + financial problems”. In terms of the lowest percentages, 76.3% of Bachelor students chose “family + love + financial problems”, whereas 14.7% of Master and PhD students chose “sleeping habits change + Higher Education transition”.

**Table 3.** Perceived Causes for Sleep Difficulties by Cycle of Studies (*N* = 456^a^).

	Bachelor *N* (%)	Master + PhD *N* (%)
Stress + academic problems	320 (77.1)	95 (22.9)
Family problems + affective/love problems + financial problems	158 (76.3)	49 (23.7)
Worries	204 (80.0)	51 (21.0)
Illness + pain	41 (82.0)	9 (18.0)
Sleeping habits change + higher education transition	122 (85.3)	21 (14.7)

*Note.* The participants could choose more than one option.

^a^It includes 5 (1.1%) missing values from participants who self-reported sleeping problems but did not choose any of the options.

### Perceived Causes for Sleep Difficulties by Insomnia Severity Groups

Regarding the severity of insomnia across the groups of perceived causes of the respondents’ sleeping difficulties (cf. Table 4), the highest proportions of clinically significant insomnia were found in the categories of “family + love + financial problems” (54.6%), “worries” (51.4%), and “illness + pain” (50%). For subthreshold insomnia, the highest percentages were observed in the “sleeping habits change + Higher Education transition” category (47.6%). However, all groups of perceived causes show similar figures, with the highest being 47.6% in “sleeping habits change + Higher Education transition” and the lowest at 40.1% in “family problems + affective/love problems + financial problems”. If we also consider the “Do not know” option in the subthreshold insomnia column, the overall figure rises to 57.1%. Finally, when examining the three severity groups of insomnia identified in Table 4, “stress + academic problems” and “sleeping habits change + Higher Education transition” show similar values: 8.2% and 7.7% (absence of insomnia), 45.1% and 47.6% (subthreshold insomnia), and 46.7% and 44.8% (moderate + severe insomnia), respectively.

**Table 4.** Perceived Causes for Sleep Difficulties by Insomnia Severity Groups (*N* = 456^a^).

	Absense of Insomnia *N* (%)	Subthreshold Insomnia *N* (%)	Moderate + Severe Insomnia *N* (%)
Stress + academic problems	34 (8.2)	187 (45.1)	194 (46.7)
Family + affective/love + financial problems	11 (5.3)	83 (40.1)	113 (54.6)
Worries	19 (7.5)	105 (41.2)	131 (51.4)
Illness + pain	3 (6.0)	22 (44.0)	25 (50.0)
Sleeping habits change + higher education transition	11 (7.7)	68 (47.6)	64 (44.8)

*Note.* The participants could choose more than one option.

^a^It includes 5 (1.1%) missing values from participants who self-reported sleeping problems but did not choose any of the options.

### Perceived Causes for Sleep Difficulties and Sleep Satisfaction/Dissatisfaction

As shown in Table 5, the most dissatisfied with their sleep were those in the group “Illness + Pain” (10.0%), followed by those in groups “family + love + financial problems” (8.2%), “sleeping habits change + Higher Education transition” (7.0%), “stress + academic problems” (6.0%), and “worries” (5.9%). Most respondents who were moderately satisfied did not know the causes of their sleeping problems (26.2%). With the exception of “sleeping habits change + Higher Education transition” (9.1%), the remaining moderately satisfied individuals were in groups with similar percentages: “family + love + financial problems” (15.9%), “stress + academic problems” (14.7%), “worries” (14.5%), and “illness + pain” (14.0%). The most satisfied with sleep were in the group “sleeping habits change + Higher Education transition” (83.9%), followed by groups “worries” (79.6%), “stress + academic problems” (79.3%), “illness + pain” (76.0%), and “family + love + financial problems” (75.8%).

**Table 5.** Perceived Causes for Sleep Difficulties and Sleep Satisfaction (*N* = 456^a^).

	Satisfied *N* (%)	Moderately Satisfied *N* (%)	Dissatisfied *N* (%)
Stress + academic problems	329 (79.3)	61 (14.7)	25 (6.0)
Family problems + affective/love problems + financial problems	157 (75.8)	33 (15.9)	17 (8.2)
Worries	203 (79.6)	37 (14.5)	15 (5.9)
Illness + pain	38 (76.0)	7 (14.0)	5 (10.0)
Sleeping habits change + higher education transition	120 (83.9)	13 (9.1)	10 (7.0)

*Note.* The participants could choose more than one option.

^a^It includes 5 (1.1%) missing values from participants who self-reported sleeping problems but did not choose any of the options.

### Perceived Causes for Sleep Difficulties by Anxiety and Depression

Tables 6 and 7 show the reasons for sleeping difficulties related to anxiety and depression. Table 6 presents the distribution of perceived causes of sleeping difficulties across three categories of anxiety and depression: absence of symptoms, mild symptoms, and moderate to severe symptoms. Regarding anxiety levels, the percentages of individuals with clinically significant anxiety (moderate to severe category) are higher than the percentages of those with lower levels of anxiety (absence of symptoms and mild categories). Among those with clinically significant anxiety, the highest percentages were observed in the following groups: “family + affective/love + financial problems” (65.2%), “illness + pain” (60%), and “worries” (58.8%).

**Table 6.** Perceived Causes of Sleep Difficulties by Anxiety (*N* = 456^a^).

	Absence of Symptoms *N* (%)	Mild *N* (%)	Moderate to Severe *N* (%)
Stress + academic problems	87 (21.0)	93 (22.4)	235 (56.6)
Family problems + affective/love problems + financial problems	23 (11.1)	49 (23.7)	135 (65.2)
Worries	44 (17.3)	61 (23.9)	150 (58.8)
Illness + pain	9 (18.0)	11 (22.0)	30 (60.0)
Sleeping habits change + higher education transition	35 (24.5)	28 (19.6)	80 (55.9)

*Note.* The participants could choose more than one option.

^a^It includes 5 (1.1%) missing values from participants who self-reported sleeping problems but did not choose any of the options.

**Table 7.** Perceived Causes of Sleep Difficulties by Depression (*N* = 456^a^).

	Absence of Symptoms *N* (%)	Mild *N* (%)	Moderate to Severe *N* (%)
Stress + academic problems	226 (54.5)	107 (25.8)	82 (19.8)
Family problems + affective/love problems + financial problems	104 (50.2)	56 (27.1)	47 (22.7)
Worries	143 (56.1)	60 (23.5)	52 (20.4)
Illness + pain	26 (52.0)	7 (14.0)	17 (34.0)
Sleeping habits change + higher education transition	87 (60.8)	31 (21.7)	25 (17.5)

*Note.* The participants could choose more than one option; *HADS-D =* Hospital Anxiety and Depression Scale-Depression.

^a^It includes 5 (1.1%) missing values from participants who self-reported sleeping problems but did not choose any of the options.

The opposite was found regarding levels of depression (cf. Table 7); individuals with no depression (absence of symptoms) had the highest percentages by far. In comparing clinically significant depression (moderate to severe category) with mild depression (mild category), the percentages for mild depression were consistently higher across all perceived causes of sleep difficulties. The only exception was in the “illness + pain group”, where the figures for clinically significant depression were higher (34%) than those for mild depression (14%). When considering “stress and academic problems”, cases of moderate to severe depression occurred in only 19.8% of instances.

## Discussion

To our knowledge, this study is the first to explore Portuguese higher education students’ perceived causes of their sleep difficulties.

In total, 30% of students reported suffering from insomnia, the same percentage as found in higher education students in Norway (Sivertsen et al., 2019). Clinically significant insomnia (ISI) was present in 17.8% students, a prevalence about the same (18.5%) as in the systematic review of Jiang et al. (2015) and close to the 13% of university students that considered to have sleep problems (Gomes et al., 2009). In our study, 12.5% students reported sleep dissatisfaction, a prevalence close to the 10.1% found in the Portuguese general population (Ohayon & Paiva, 2005).

Among the participants in this study, the prevalence of anxiety was (38.86%) which is higher than the pooled prevalence (32%) found during the COVID-19 pandemic (cf. meta-analysis by Deng et al., 2021). On the other hand, in that meta-analysis, the pooled prevalence of depressive symptoms was much higher at 34% compared to the prevalence found in our study, which was 9.3%. However, Deng et al. (2021) found that values differ based on geographical regions, diagnostic criteria, education level, undergraduate year of study, financial situation, and living arrangements.

On average, students selected three reasons for their sleeping problems, the most common combinations being “perceived stress + family problems”, “stress + academic problems + family problems”, and stress.

The top three reasons students considered most important for their sleep difficulties were stress (85%), academic/study problems (67%) and worries in general (56.5%). In the general population, the most commonly attributed cause of disturbed sleep was stress (35.1%) in the study of Petersen et al. (2022) and worry/overthinking in the study of Armstrong and Dregan (2014).

Research with university students showed that the prevalence of sleep disturbances attributed to worry was about 33% (Marques et al., 2016), a figure lower than that found in the present study. Methodological differences such as sample characteristics, data collection (data were collected during classes in the academic years 2007–2008 and 2008–2009), and the question used may, in part, explain the difference between the two studies. However, there may also have been an increase in “anxious insomnia”. First, a retrospective study revealed a steady increase in students worry levels (pathological worrying) over two decades (Davey et al., 2022). Second, a recent paper found that about 50% of Portuguese students reported that worrying is a problem that bothers them, and 12.1% mentioned being worried with such intensity that “they cannot think or do anything else” (Reis et al., 2019). Third, worry is a common factor between Generalized Anxiety Disorder and those with sleep problems such as insomnia (Harvey, 2009). Fourth, in the current study, those students who attributed their sleep difficulties to worry (58.8%) had clinical anxiety and 51.4% had clinically significant insomnia. Marques et al. (2016) also found that pre-sleep cognitive arousal, perceived academic stress, arousability, tendency to worry, and perceived physical health were significant predictors of sleep disturbance due to worry.

In the current study, it was found that sleep disturbances due to worries decreased with age and level of education. The highest prevalence (53.3%) was observed in the 18–20 age group, compared to 9% in the 25–30 age group. Additionally, 80% of individuals pursuing Bachelorʼs degrees reported sleep disturbances, whereas only 21% of those in Masterʼs or PhD programs reported the same. This suggests that as individuals mature, their coping strategies evolve towards more adaptive styles. It highlights the importance of introducing prevention and intervention programs early in young adulthood to enhance coping capabilities (Jenzer et al., 2019) and, in turn, to ensure better sleep quality.

In line with the literature mentioned in the introduction, results of the present study show a high proportion of students attributing their sleep disturbances to love (28.4%) and family problems (27.5%), with a smaller percentage related to financial problems (13.7%).

An important and new finding of this study is the rather high prevalence (25.9%) of sleep difficulties attributed to “changes in sleeping habits”, whereas 10.4% respondents selected sleep difficulties due to “transition to higher education”. A previous study involving college students found that regular sleepers had better mood and psychomotor performance, as well as increased time spent in REM and slow-wave sleep (Medeiros et al., 2001). Additionally, another prospective study with college students showed that both consistent bedtimes and wake times were associated with improved sleep quality (Carney et al., 2006). Furthermore, maintaining a consistent sleep schedule contribute to healthy sleep outcomes (Chaput et al., 2020). In this context, it worth mention that regularity of sleep is a core component of good sleep health (Buysse, 2014).

As a group, “changes in sleeping habits + higher education transition” was most prominent in the age range of 18–20 years, particularly among Bachelor’s students (85.3%). Their reported levels of anxiety were approximately 56%, with about 45% experiencing clinical insomnia and around 48% experiencing sub-clinical insomnia.

As expected in a young population (Armstrong & Dregan, 2014; Petersen et al., 2022) pain (7.1%) and illness in general (5.5%) were the least frequent reasons respondents provided for their sleep problems. Regarding pain, our percentage is similar to the 8% found by Lund et al. (2010) in university students. As a group, “illness in general + pain” was highest in the younger age group, with 48% reporting this. Additionally, 50% experienced clinical insomnia, and 60% had clinical and anxiety concomitantly.

Concerning the connection between health and sleep, studies have shown that sleep quality was the stronger and more consistent predictor of mental and physical health (Gomes, 2005; Pilcher et al., 1997). In addition, students classified as poor quality sleepers reported significantly more problems with physical and psychological health than did good quality sleepers (Lund et al., 2010). Additionally, studies on university students in the United Kingdom and Egypt found that perceived stress was highly and significantly associated with psychological symptoms (e.g., nervousness/anxiety, depressive mood, difficulties to concentrate, sleep disorder/insomnia, etc.,) and pains/aches (back pain, neck and shoulder pain, fatigue, headaches) and with pain and aches symptoms in both countries (El Ansari et al., 2014). They also found that those participants perceiving their health as fair/poor had consistently higher ratings across both psychological and pains/aches complaints.

Overall, our findings align with the cognitive model of insomnia, which posits that a predisposition to worry in response to stress is a key factor contributing to insomnia. This tendency may lead to increased arousal before sleep, which can, in turn, disrupt sleep and result in insomnia (Perlis et al., 2011). Moreover, Ellis and Cropley (2002) found that worry as a maladaptive coping style was associated with both self-defined acute and chronic insomnia suggesting that worry may both serve as a predisposing and precipitating factor for insomnia. Additionally, a prospective study supports the view that worry contributes to the maintenance of sleep problem and the development of chronic insomnia (Jansson & Linton, 2006). Moreover, proneness to worry is very prevalent in students (Marques et al., 2016) and worry is considered a transdiagnostic process across anxiety and insomnia conditions. Therefore, interventions aimed at reducing the levels of worry may be beneficial for preventing poor mental health and promoting well-being in young adults (Bell et al., 2023; Clancy et al., 2020; Edge et al., 2021).

In our sample, about 9% of the students did not identify a cause for their sleep difficulties; this is a number close to that found in epidemiologic studies of insomnia (12–16%) in the general population (Ohayon, 2011), but much lower than the one found (31%) in a study comprising UK adult population aged 16–74 years (Armstrong & Dregan, 2014). In a study of primary care patients, about 6% responded that their sleep was disturbed but did not know the reason or did not attribute the disturbance to any particular factor, perhaps it is “*my nature*”, some said (Azevedo, 1989). Prior to DSM-5, these cases received the diagnosis of “primary insomnia” but DSM-5 removed it in favour of “insomnia disorder” (American Psychiatric Association, 2013).

To some people, not knowing the cause of their insomnia is perplexing, even more so when his/her doctor do not know as well. As observed by a student with sleep onset problems “*I go to a psychiatrist and he doesn’t even understand why. Environmental factor? I am on medication*” (Gomes, 2005, p. 521). Research of what characterizes these individuals is needed in order to help patients and physicians make informed decisions. Finally, the fact that, in the open question, none of the respondents described other reasons for their sleep problems may reflect the fact that the options provided covered all types of potential causes for this sample.

Our study presents some limitations. First, despite the advantages of online data collection, there were challenges in controlling the study. There may have been a greater number of potential duplicate submissions, and there could be a higher representation of participants who answered the questionnaire purely out of interest in the subject of sleep. This might have led to an overestimation of the reported difficulties. Second, data collection occurred during SARS-CoV-2 pandemic, which may have had a significant impact on results. Third, due to the skewed gender-distribution (76.9% women), it is unclear if this sex disproportion affected the study᾽s results as observed in the work by Sivertsen et al., 2019). However, we should outline that the percentage of women is high because they represent the most part of the higher education students in Portugal (OECD, 2022) and they are more prone to sleep problems as observed in various studies (e.g., Zeng et al., 2020). Even so, future investigations should include a more balanced sample in terms of sex and analyze men and women separately. Fourth, a major limitation is that given that the responses provided by participants were not mutually exclusive, we could not perform inferential statistics. However, in the real world of clinical practice individuals seldom give one single factor for their sleep problems. Future studies, could “force” individuals to choose the most “of the most important” among the causes they give. Lastly, it would be interesting to correlate our results with patterns of drug consumption, including coffee, prescribed medication, and over-the-counter medications. Strengths of the current study include the large sample size, diverse field and cycle of studies, and the use of standard measures of insomnia and mental health (anxiety, depression). One notable strength of the study is the exploration of a wide range of causal attributions of sleep problems simultaneously, in a field mostly based in correlational/association studies.

## Conclusion

Overall, this study indicates a broad range of difficulties experienced by many students that all stakeholders should pay attention, in order to build a high-quality learning environment (Rusticus et al., 2023). Our findings suggest that prevention and intervention programs should consider: a) stress management interventions for stressed students (Amanvermez et al., 2023, 2022); b) intervention strategies focusing on worry, not only to reduce worry but also to improve sleep quality and physical health (McCarrick et al., 2021); c) psychological and behavioural interventions to improve sleep health (Friedrich & Schlarb, 2018; Griggs et al., 2020); d) interventions aimed to prepare students and their families, for a successful transition from high school to higher education (Cage et al., 2021), and e) sleep education programs to improve knowledge on sleep, sleep behaviours (e.g., waking earlier during the week, maintaining a more regular sleep schedule, and reducing naps), sleep quality and mood (Hershner & O’Brien, 2018).

Finally, it is noteworthy to mention that, despite the effectiveness of these non-pharmacological interventions in improving student health and wellbeing, only a very small number of students receive psychological treatment for their sleep problems. (Silva et al., 2021).

## Data Availability

Share upon reasonable request.*

## References

[bibr1-00332941241299730] Adams G. C. Stoops M. A. Skomro R. P. (2014). Sleep tight: Exploring the relationship between sleep and attachment style across the life span. Sleep Medicine Reviews, 18(6), 495–507. 10.1016/j.smrv.2014.03.002 24721278

[bibr2-00332941241299730] Amanvermez Y. Rahmadiana M. Karyotaki E. de Wit L. Ebert D. D. Kessler R. C. Cuijpers P. (2023). Stress management interventions for college students: A systematic review and meta-analysis. Clinical Psychology: Science and Practice,30(4), 423–444, Article e12342. 10.1111/cpsp.12342

[bibr3-00332941241299730] Amanvermez Y. Zhao R. Cuijpers P. de Wit L. M. Ebert D. D. Kessler R. C. Bruffaerts R. Karyotaki E. (2022). Effects of self-guided stress management interventions in college students: A systematic review and meta-analysis. Internet Interventions, 28, 100503. 10.1016/j.invent.2022.100503 35242591 PMC8861419

[bibr4-00332941241299730] American Psychiatric Association . (2013). Diagnostic and statistical manual of mental disorders-5 (5th ed.). American Psychiatric Association.

[bibr5-00332941241299730] Araújo T. Jarrin D. C. Leanza Y. Vallières A. Morin C. M. (2017). Qualitative studies of insomnia: Current state of knowledge in the field. Sleep Medicine Reviews, 31, 58–69. 10.1016/j.smrv.2016.01.003 27090821 PMC4945477

[bibr6-00332941241299730] Armstrong D. Dregan A. (2014). A population-based investigation into the self-reported reasons for sleep problems. PLoS One, 9(7), Article e101368. 10.1371/journal.pone.0101368 24983754 PMC4077805

[bibr7-00332941241299730] Arnett J. J. Žukauskienė R. Sugimura K. (2014). The new life stage of emerging adulthood at ages 18–29 years: Implications for mental health. The Lancet Psychiatry, 1(7), 569–576. 10.1016/S2215-0366(14)00080-7 26361316

[bibr8-00332941241299730] Azevedo M. H. (1989). Avaliação subjectiva do sono-vigília e fenomenologia da insónia. Lição Síntese. Provas de Agregação em Psiquiatria. Faculdade de Medicina, Universidade de Coimbra.

[bibr9-00332941241299730] Azevedo M. H. Bos S. C. Soares M. J. Marques M. Pereira A. T. Maia B. Gomes A. A. Macedo A. (2010). Longitudinal study on perfectionism and sleep disturbance. World Journal of Biological Psychiatry, 11(2 Pt 2), 476–485. 10.3109/15622970903304467 20218803

[bibr10-00332941241299730] Bajoghli H. Keshavarzi Z. Mohammadi M. R. Schmidt N. B. Norton P. J. Holsboer-Trachsler E. Brand S. (2014). “I love you more than I can stand!” - romantic love, symptoms of depression and anxiety, and sleep complaints are related among young adults. International Journal of Psychiatry in Clinical Practice, 18(3), 169–174. 10.3109/13651501.2014.902072 24611539

[bibr11-00332941241299730] Bei B. Wiley J. F. Trinder J. Manber R. (2016). Beyond the mean: A systematic review on the correlates of daily intraindividual variability of sleep/wake patterns. Sleep Medicine Reviews, 28, 108–124. 10.1016/j.smrv.2015.06.003 26588182

[bibr12-00332941241299730] Bell I. H. Marx W. Nguyen K. Grace S. Gleeson J. Alvarez-Jimenez M. (2023). The effect of psychological treatment on repetitive negative thinking in youth depression and anxiety: A meta-analysis and meta-regression. Psychological Medicine, 53(1), 6–16. 10.1017/S0033291722003373 36373473 PMC9875014

[bibr13-00332941241299730] Bjelland I. Dahl A. A. Haug T. T. Neckelmann D. (2002). The validity of the Hospital Anxiety and Depression Scale. An updated literature review. Journal of Psychosomatic Research, 52(2), 69–77. 10.1016/s0022-3999(01)00296-3 11832252

[bibr14-00332941241299730] Bjørnnes A. K. Torbjørnsen A. Valeberg B. T. Sparboe-Nilsen B. B. Sandbekken I. H. Almendingen K. Leegaard M. Ravn I. Sæterstrand M. T. Løyland B. Kvarme L. G. Fagerlund B. H. Valla L. Misvær N. Riiser K. Utne I. Rostad H. Winger A. Albertini Früh E. Grov E. K. (2021). What is known about students and sleep: Systematic review and evidence map. Sage Open, 11(3). 10.1177/21582440211032162

[bibr15-00332941241299730] Buysse D. J. (2014). Sleep health: Can we define it? Does it matter? Sleep, 37(1), 9–17. 10.5665/sleep.3298 24470692 PMC3902880

[bibr16-00332941241299730] Cabrita J. Ferreira H. Iglésias P. Baptista T. Rocha E. Lopes da Silva A. Pereira Miguel J. (2004). Patterns and determinants of psychoactive drug use in Lisbon University students - a population-based study. Pharmacy World and Science, 26(2), 79–82. 10.1023/b:phar.0000018597.46246.34 15085941

[bibr17-00332941241299730] Cage E. Jones E. Ryan G. Hughes G. Spanner L. (2021). Student mental health and transitions into, through and out of university: Student and staff perspectives. Journal of Further and Higher Education, 45(8), 1076–1089. 10.1080/0309877X.2021.1875203

[bibr18-00332941241299730] Carney C. E. Edinger J. D. Meyer B. Lindman L. Istre T. (2006). Daily activities and sleep quality in college students. Chronobiology International, 23(3), 623–637. 10.1080/07420520600650695 16753946

[bibr19-00332941241299730] Chaput J. P. Dutil C. Featherstone R. Ross R. Giangregorio L. Saunders T. J. Janssen I. Poitras V. J. Kho M. E. Ross-White A. Zankar S. Carrier J. (2020). Sleep timing, sleep consistency, and health in adults: A systematic review. Applied Physiology Nutrition and Metabolism, 45(10 (Suppl. 2)), S232–S247. 10.1139/apnm-2020-0032 33054339

[bibr20-00332941241299730] Clancy F. Prestwich A. Caperon L. Tsipa A. O’Connor D. B. (2020). The association between worry and rumination with sleep in non-clinical populations: A systematic review and meta-analysis. Health Psychology Review, 14(4), 427–448. 10.1080/17437199.2019.1700819 31910749

[bibr21-00332941241299730] Clemente V. Ruivo Marques D. Miller-Mendes M. Morin C. M. Serra J. Allen Gomes A. (2021). The European Portuguese version of the insomnia severity index. Journal of Sleep Research, 30(1), Article e13198. 10.1111/jsr.13198 32997368

[bibr22-00332941241299730] Davey G. C. L. Meeten F. Field A. P. (2022). Whatʼs worrying our students? Increasing worry levels over two decades and a new measure of student worry frequency and domains. Cognitive Therapy and Research, 46(2), 406–419. 10.1007/s10608-021-10270-0 34658461 PMC8501938

[bibr23-00332941241299730] Deng J. Zhou F. Hou W. Silver Z. Wong C. Y. Chang O. Drakos A. Zuo Q. K. Huang E. (2021). The prevalence of depressive symptoms, anxiety symptoms and sleep disturbance in higher education students during the COVID-19 pandemic: A systematic review and meta-analysis. Psychiatry Research, 301, 113863. 10.1016/j.psychres.2021.113863 33984824 PMC9225824

[bibr24-00332941241299730] Edge D. Newbold A. Ehring T. Rosenkranz T. Frost M. Watkins E. R. (2021). Reducing worry and rumination in young adults via a mobile phone app: Study protocol of the ECoWeB (emotional competence for well-being in young adults) randomised controlled trial focused on repetitive negative thinking. BMC Psychiatry, 21(1), 519. 10.1186/s12888-021-03536-0 34674669 PMC8532278

[bibr25-00332941241299730] El Ansari W. Oskrochi R. Haghgoo G. (2014). Are students’ symptoms and health complaints associated with perceived stress at university? Perspectives from the United Kingdom and Egypt. International Journal of Environmental Research and Public Health, 11(10), 9981–10002. 10.3390/ijerph111009981 25264677 PMC4210962

[bibr26-00332941241299730] Ellis J. Cropley M. (2002). An examination of thought control strategies employed by acute and chronic insomniacs. Sleep Medicine, 3(5), 393–400. 10.1016/s1389-9457(02)00039-4 14592170

[bibr27-00332941241299730] Fernandez-Mendoza J. Lenker K. P. Calhoun S. L. Qureshi M. Ricci A. Bourchtein E. He F. Vgontzas A. N. Liao J. Liao D. Bixler E. O. (2022). Trajectories of insomnia symptoms from childhood through young adulthood. Pediatrics, 149(3), Article e2021053616. 10.1542/peds.2021-053616 35174394 PMC8900485

[bibr28-00332941241299730] Friedrich A. Schlarb A. A. (2018). Let’s talk about sleep: A systematic review of psychological interventions to improve sleep in college students. Journal of Sleep Research, 27(1), 4–22. 10.1111/jsr.12568 28618185

[bibr29-00332941241299730] Galambos N. L. Dalton A. L. Maggs J. L. (2009). Losing sleep over it: Daily variation in sleep quantity and quality in Canadian students’ first semester of university. Journal of Research on Adolescence, 19(4), 741–761. 10.1111/j.1532-7795.2009.00618.x

[bibr30-00332941241299730] García-Ros R. Pérez-González F. Pérez-Blasco J. Natividad L. A. (2012). Evaluación del estrés académico en estudiantes de nueva incorporación a la universidad. Revista Latinoamericana De Psicologia, 44(2), 143–154. https://www.redalyc.org/articulo.oa?id=80524058011.

[bibr31-00332941241299730] Gardani M. Bradford D. R. R. Russell K. Allan S. Beattie L. Ellis J. G. Akram U. (2022). A systematic review and meta-analysis of poor sleep, insomnia symptoms and stress in undergraduate students. Sleep Medicine Reviews, 61, 101565. 10.1016/j.smrv.2021.101565 34922108

[bibr32-00332941241299730] Gellis L. A. Park A. Stotsky M. T. Taylor D. J. (2014). Associations between sleep hygiene and insomnia severity in college students: Cross-sectional and prospective analyses. Behavior Therapy, 45(6), 806–816. 10.1016/j.beth.2014.05.002 25311289

[bibr33-00332941241299730] Gomes A. A. (2005). Sono, sucesso académico e bem-estar em estudantes universitários [Sleep, academic success, and wellbeing in undergraduates]. PhD Dissertation. University of Aveiro. https://ria.ua.pt/handle/10773/1103

[bibr34-00332941241299730] Gomes A. A. Tavares J. Azevedo M. H. P. (2009). Padrões de sono em estudantes universitários portugueses/sleep-wake patterns in Portuguese undergraduates. Acta Medica Portuguesa, 22(5), 545–552. https://www.actamedicaportuguesa.com/revista/index.php/amp/article/view/1719 19944037

[bibr35-00332941241299730] Griggs S. Conley S. Batten J. Grey M. (2020). A systematic review and meta-analysis of behavioral sleep interventions for adolescents and emerging adults. Sleep Medicine Reviews, 54, 101356. 10.1016/j.smrv.2020.101356 32731152 PMC7669566

[bibr36-00332941241299730] Harvey A. G. (2009). A transdiagnostic approach to treating sleep disturbance in psychiatric disorders. Cognitive Behavior Therapy, 38(Suppl 1), 35–42. 10.1080/16506070903033825 19697179

[bibr37-00332941241299730] Hershner S. O’Brien L. M. (2018). The impact of a randomized sleep education intervention for college students. Journal of Clinical Sleep Medicine, 14(03), 337–347. 10.5664/jcsm.6974 29510791 PMC5837835

[bibr38-00332941241299730] Jansson M. Linton S. J. (2006). The development of insomnia within the first year: A focus on worry. British Journal of Health Psychology, 11(Pt 3), 501–511. 10.1348/135910705X57412 16870058

[bibr83-00332941241299730] Jenzer T. Read J. P. Naragon-Gainey K. Prince M. A. (2019). Coping trajectories in emerging adulthood: The influence of temperament and gender. Journal of Personality, 87(3), 607–619. 10.1111/jopy.12419 29999532 PMC6330139

[bibr39-00332941241299730] Jiang X. L. Zheng X. Y. Yang J. Ye C. P. Chen Y. Y. Zhang Z. G. Xiao Z. J. (2015). A systematic review of studies on the prevalence of insomnia in university students. Public Health, 129(12), 1579–1584. 10.1016/j.puhe.2015.07.030 26298588

[bibr40-00332941241299730] Karyotaki E. Cuijpers P. Albor Y. Alonso J. Auerbach R. P. Bantjes J. Bruffaerts R. Ebert D. D. Hasking P. Kiekens G. Lee S. McLafferty M. Mak A. Mortier P. Sampson N. A. Stein D. J. Vilagut G. Kessler R. C. (2020). Sources of stress and their associations with mental disorders among college students: Results of the world health organization world mental health surveys international college student initiative. Frontiers in Psychology, 11, 1759. 10.3389/fpsyg.2020.01759 32849042 PMC7406671

[bibr41-00332941241299730] Lund H. G. Reider B. D. Whiting A. B. Prichard J. R. (2010). Sleep patterns and predictors of disturbed sleep in a large population of college students. Journal of Adolescent Health, 46(2), 124–132. 10.1016/j.jadohealth.2009.06.016 20113918

[bibr42-00332941241299730] Marques D. R. Gomes A. A. Ferreira M. F. de Azevedo M. H. P. (2016). Don't worry, sleep well: Predictors of sleep loss over worry. Sleep and Biological Rhythms, 14(3), 309–318. 10.1007/s41105-016-0060-z

[bibr43-00332941241299730] McCarrick D. Prestwich A. Prudenzi A. O’Connor D. B. (2021). Health effects of psychological interventions for worry and rumination: A meta-analysis. Health Psychology, 40(9), 617–630. 10.1037/hea0000985 34410760

[bibr44-00332941241299730] Medeiros A. L. Mendes D. B. Lima P. F. Araujo J. F. (2001). The relationships between sleep-wake cycle and academic performance in medical students. Biological Rhythm Research, 32(2), 263–270. 10.1076/brhm.32.2.263.1359

[bibr45-00332941241299730] Morin C. M. Belleville G. Bélanger L. Ivers H. (2011). The Insomnia Severity Index: Psychometric indicators to detect insomnia cases and evaluate treatment response. Sleep, 34(5), 601–608. 10.1093/sleep/34.5.601 21532953 PMC3079939

[bibr46-00332941241299730] OECD . (2022). Education at a glance 2022: OECD indicators. OECD Publishing. 10.1787/3197152b-en

[bibr47-00332941241299730] Ohayon M. M. (2011). Epidemiological overview of sleep disorders in the general population. Sleep Medicine Research, 2(1), 1–9. 10.17241/smr.2011.2.1.1

[bibr48-00332941241299730] Ohayon M. M. Paiva T. (2005). Global sleep dissatisfaction for the assessment of insomnia severity in the general population of Portugal. Sleep Medicine, 6(5), 435–441. 10.1016/j.sleep.2005.03.006 16085459

[bibr49-00332941241299730] Ohayon M. M. Reynolds C. F., 3rd. (2009). Epidemiological and clinical relevance of insomnia diagnosis algorithms according to the DSM-IV and the International Classification of Sleep Disorders (ICSD). Sleep Medicine, 10(9), 952–960. 10.1016/j.sleep.2009.07.008 19748312 PMC3715324

[bibr50-00332941241299730] Pais-Ribeiro J. Silva I. Ferreira T. Martins A. Meneses R. Baltar M. (2007). Validation study of a Portuguese version of the hospital anxiety and depression scale. Psychology Health & Medicine, 12(2), 225–237. 10.1080/13548500500524088 17365902

[bibr51-00332941241299730] Pascoe M. C. Hetrick S. E. Parker A. G. (2020). The impact of stress on students in secondary school and higher education. International Journal of Adolescence and Youth, 25(1), 104–112. 10.1080/02673843.2019.1596823

[bibr52-00332941241299730] Peltz J. S. Bodenlos J. S. Kingery J. N. Rogge R. D. (2021). The role of financial strain in college students’ work hours, sleep, and mental health. Journal of American College Health, 69(6), 577–584. 10.1080/07448481.2019.1705306 31940259

[bibr53-00332941241299730] Perlis M. Shaw P. J. Cano G. Espie C. A. (2011). Models of insomnia. In Kryger M. H. Roth T. Dement W. C. (Eds.), Principles and practice of sleep medicine (5 ed., pp. 850–865). Elsevier Saunders.

[bibr54-00332941241299730] Petersen H. Kecklund G. Åkerstedt T. (2022). Disturbed sleep and its attribution to stress and other causes: A population-based survey. Scandinavian Journal of Psychology, 64(2), 99–104. Advance online publication. 10.1111/sjop.12867 36057792

[bibr55-00332941241299730] Pilcher J. J. Ginter D. R. Sadowsky B. (1997). Sleep quality versus sleep quantity: Relationships between sleep and measures of health, well-being and sleepiness in college students. Journal of Psychosomatic Research, 42(6), 583–596. 10.1016/s0022-3999(97)00004-4 9226606

[bibr56-00332941241299730] Reis M. Ramiro L. Gaspar de Matos M. (2019). Worries, mental and emotional health difficulties of Portuguese university students. Advances in Social Sciences Research Journal, 6(7), 558–569. 10.14738/assrj.67.6818

[bibr57-00332941241299730] Robotham D. (2008). Stress among higher education students: Towards a research agenda. Higher Education, 56(6), 735–746. 10.1007/s10734-008-9137-1

[bibr58-00332941241299730] Rusticus S. A. Pashootan T. Mah A. (2023). What are the key elements of a positive learning environment? Perspectives from students and faculty. Learning Environments Research, 26(1), 161–175. 10.1007/s10984-022-09410-4 35574193 PMC9076804

[bibr59-00332941241299730] Silva J. Vieira P. Gomes A. A. Roth T. de Azevedo M. H. Marques D. R. (2021). Sleep difficulties and use of prescription and non-prescription sleep aids in Portuguese higher education students. Sleep Epidemiology, 1, 100012. 10.1016/j.sleepe.2021.100012

[bibr60-00332941241299730] Sivertsen B. Hysing M. Harvey A. G. Petrie K. J. (2021). The Epidemiology of insomnia and sleep duration across mental and physical health: The shot study. Frontiers in Psychology, 12, 662572. 10.3389/fpsyg.2021.662572 34194368 PMC8236531

[bibr61-00332941241299730] Sivertsen B. Vedaa Ø. Harvey A. G. Glozier N. Pallesen S. Aarø L. E. Lønning K. J. Hysing M. (2019). Sleep patterns and insomnia in young adults: A national survey of Norwegian university students. Journal of Sleep Research, 28(2), Article e12790. 10.1111/jsr.12790 30515935

[bibr62-00332941241299730] Snaith R. P. (2003). The hospital anxiety and depression scale. Health and Quality of Life Outcomes, 1(29), 29–394. 10.1186/1477-7525-1-29 12914662 PMC183845

[bibr63-00332941241299730] Stewart M. A. (1995). Effective physician-patient communication and health outcomes: A review. Canadian Medical Association Journal, 152(9), 1423–1433. 7728691 PMC1337906

[bibr64-00332941241299730] Talero-Gutierrez C. Duran-Torres F. Ibañez-Pinilla M. Perez-Olmos I. Echeverria-Palacio C. M. (2017). Sleep quality perception and romantic relationships in university students: Cross-sectional study. Revista de la Facultad de Medicina, 65(2), 197–202. 10.15446/revfacmed.v65n3.58396

[bibr65-00332941241299730] Tsai L. L. Li S. P. (2004). Sleep patterns in college students: Gender and grade differences. Journal of Psychosomatic Research, 56(2), 231–237. 10.1016/S0022-3999(03)00507-5 15016583

[bibr66-00332941241299730] Zeng L. N. Zong Q. Q. Yang Y. Zhang L. Xiang Y. F. Ng C. H. Chen L. G. Xiang Y. T. (2020). Gender difference in the prevalence of insomnia: A meta-analysis of observational studies. Frontiers in Psychiatry, 11, 577429. 10.3389/fpsyt.2020.577429 33329116 PMC7714764

[bibr67-00332941241299730] Zhou J. Qu J. Ji S. Bu Y. Hu Y. Sun H. Xue M. Zhou T. Qu J. Liu Y. (2022). Research trends in college students’ sleep from 2012 to 2021: A bibliometric analysis. Frontiers in Psychiatry, 13, 1005459. 10.3389/fpsyt.2022.1005459 36203831 PMC9530190

[bibr68-00332941241299730] Zigmond A. S. Snaith R. P. (1983). The hospital anxiety and depression scale. Acta Psychiatrica Scandinavica, 67(6), 361–370. 10.1111/j.1600-0447.1983.tb09716.x 6880820

